# Metabolome Profiling of Yokukansan in Preventing Postoperative Delirium in Elderly Cancer Patients: A Reverse Translational Study

**DOI:** 10.1111/pcn.13875

**Published:** 2025-08-05

**Authors:** Ryoichi Sadahiro, Katsuya Ohbuchi, Taiki Nakaya, Sei Manabe, Saho Wada, Takuhiro Yamaguchi, Masahiro Sugimoto, Ken Shimizu, Tetsufumi Sato, Minoru Esaki, Hiroyuki Daiko, Seiichi Yoshimoto, Yukihide Kanemitsu, Akira Kawai, Mitsuya Ishikawa, Yoshiyuki Matsui, Kazunori Aoki, Takao Ueno, Hiromichi Matsuoka, Yasuhito Uezono

**Affiliations:** ^1^ Department of Psycho‐Oncology National Cancer Center Hospital Tokyo Japan; ^2^ Department of Immune Medicine National Cancer Center Research Institute Tokyo Japan; ^3^ Tsumura Advanced Technology Research Laboratories, Tsumura & Co. Ibaraki Japan; ^4^ Department of Anesthesiology and Resuscitology Okayama University Hospital Okayama Japan; ^5^ Division of Quality Assurance Programs Institute for Cancer Control, National Cancer Center Tokyo Japan; ^6^ Division of Biostatistics Tohoku University Graduate School of Medicine Tokyo Japan; ^7^ Institute for Advanced Biosciences Keio University Yamagata Japan; ^8^ Institute of Medical Sciences Tokyo Medical University Tokyo Japan; ^9^ Department of Psycho‐Oncology Cancer Institute Hospital of Japanese Foundation for Cancer Research Tokyo Japan; ^10^ Department of Anesthesiology and Intensive Care Medicine National Cancer Center Hospital Tokyo Japan; ^11^ Department of Hepatobiliary and Pancreatic Surgery National Cancer Center Hospital Tokyo Japan; ^12^ Department of Esophageal Surgery National Cancer Center Hospital Tokyo Japan; ^13^ Department of Head and Neck Surgery National Cancer Center Hospital Tokyo Japan; ^14^ Department of Colorectal Surgery National Cancer Center Hospital Tokyo Japan; ^15^ Department of Musculoskeletal Oncology and Rehabilitation National Cancer Center Hospital Tokyo Japan; ^16^ Department of Gynecology National Cancer Center Hospital Tokyo Japan; ^17^ Department of Urology National Cancer Center Hospital Tokyo Japan; ^18^ Department of Dentistry National Cancer Center Hospital Tokyo Japan; ^19^ Department of Pain Control Research The Jikei University School of Medicine Tokyo Japan

**Keywords:** cancer surgery, metabolome profile, postoperative delirium, reverse translational study, Yokukansan

## Abstract

**Aims:**

Postoperative delirium (PD) is a common and severe complication in older adult patients undergoing invasive cancer resections. This study explored the plasma metabolome associated with PD and evaluated the efficacy of Yokukansan (YKS), a traditional Japanese Kampo medicine, in preventing PD.

**Methods:**

An ancillary study was conducted alongside a double‐blind, placebo‐controlled randomized clinical trial involving patients 65 years and older, focusing on patients older than 75 years as a primary analysis population. Plasma samples were analyzed using targeted and nontargeted metabolomics. An in vivo study using aged mice assessed the effects of YKS on plasma and brain metabolites.

**Results:**

A total of 83 patients, including 21 patients older than 75 years, were enrolled. Patients with PD had lower levels of several lipid mediators, free fatty acids, and phospholipids. YKS administration led to increased nine phospholipids and four hydrophilic metabolites in patients older than 75 years, including phosphatidylcholine (40:7) and phosphatidylcholine (42:8), which were also altered in delirium patients. In the patients older than 65 years, only two metabolites increased in the YKS administration group. In aged mice, YKS elevated plasma phospholipids, similar to findings in patients older than 75 years, and influenced brain citrulline and creatine, which related to oxidative stress and cognitive function. Correlation analyses revealed associations between plasma and brain metabolite changes.

**Conclusion:**

Our findings suggest that the plasma metabolome provides insight into the pathophysiology of PD and the potential mechanism underlying the preventive effect of YKS against PD.

In the hyper‐aged society, the number of cancer resections in older adults is rising, and such resections carry a high risk of delirium; moreover, the number of patients with delirium is expected to increase as society ages.^1^ Delirium is particularly common after highly invasive surgery^2^ and impacts postoperative management and long‐term prognosis (e.g. dementia onset and decreased autonomy).^3^ Recently, delirium has been shown to increase the risk of developing dementia, which highlights its clinical importance.^4^, ^5^ There is an urgent need to develop mechanism‐based prevention methods for postoperative delirium (PD) to improve the quality of life of patients following cancer surgery. Specifically, as we gain a better understanding of the mechanisms underlying PD,^6^ it has become crucial to understand the heterogeneity of PD^7^ and develop personalized PD prevention methods.^8^

Various metabolites have been implicated in the pathogenesis of delirium. Large‐scale studies using three genome‐wide association studies conducted on the metabolome have shown that high concentrations of the blood metabolites, low‐density lipoprotein cholesterol, and sphingomyelin (SM), and low concentrations of X‐11593‐O‐methylascorbate are causal mediators in the development of delirium.^9^ Neurotoxic quinolinic acid from the kynurenine pathway is elevated in the cerebrospinal fluid and blood of patients with delirium.^10^ The phenotype of delirium varies depending on precipitating factors.^11^ The pathophysiological mechanisms are diverse and heterogeneous.^12^ Therefore, the metabolites involved in delirium may differ depending on the precipitating factor of delirium. For example, delirium associated with coronavirus disease infection is associated with foodborne molecules in the cerebrospinal fluid.^13^ Regarding, phospholipids including phosphatidylinositol, phosphatidylcholine (PC) and SM are downregulated in patients with cardiac PD,^14^ and PD after orthopedic surgery is associated with various metabolites, including lower concentrations of free fatty acids and higher concentrations of polyamines in the blood and cerebrospinal fluid.^15^, ^16^, ^17^, ^18^, ^19^ Previous studies on noncardiac surgery have reported that disturbances in energy metabolism and amino acid synthesis pathways may contribute to an increased risk of PD.^20^, ^21^ However, the metabolites related to delirium following highly invasive cancer resection, which carries a high risk of delirium, have not yet been identified.

In a previous study, we examined the efficacy of Yokukansan (YKS, Yi‐gan san), an integrative medicine (i.e. traditional herbal medicine), which is known to act on serotonin (5‐hydroxytryptamine [5‐HT]) release, glutamate transport, and gamma‐aminobutyric acid receptor stimulation,^22^, ^23^ and induce antipsychotic effects,^24^ in preventing delirium in a double‐blind, placebo‐controlled randomized controlled trial (RCT).^25^ We showed in a secondary analysis that YKS effectively prevents delirium with hyperactive behavior in adults older than 75 years.^26^ Previous studies have shown that Chinese herbal medicine alters endogenous metabolites. In humans, Hachimigiogan, which has an adjuvant effect on acetylcholinesterase inhibitors and slows the progression of cognitive dysfunction in female patients with mild Alzheimer disease, has been shown to alter hippuric acid, phenylacetic acid, suberic acid, tartaric acid, 2‐aminooctanoic acid, and 3‐phosphoglyceric acid concentrations.^27^ We also revealed that administering Maoto, which induces antipyretic activity, decreases branched‐chain amino acid concentrations and increases the concentrations of various types of ω‐3 fatty acids.^28^ We also investigated Maoto‐derived metabolites in the blood using the novel differential annotation of converted metabolites (DAC‐Met) strategy, which is an annotation method that uses mass differences in major metabolic reactions among detected peaks and differential network analysis.^29^ Although YKS may alter certain metabolites and show efficacy in preventing PD, similarly to the aforementioned herbal medicines, this has not yet been clarified. It is necessary to verify both drug efficacy and the physiological mechanism in clinical trials to elucidate the physiological mechanism underlying the efficacy of YKS in treating diseases. However, to date, few studies of Kampo have been conducted.

The present study was a reverse translational study consisting of an ancillary study of a clinical trial and an in vivo study. As an ancillary study to a double‐blind, placebo‐controlled RCT, we explored metabolites related to: (1) delirium following highly invasive cancer resection in the placebo group, (2) YKS, by comparing the YKS group with the placebo group, and (3) the efficacy of YKS in preventing PD in the YKS group. In addition, we examined the effects of YKS on brain metabolites, controlling for individual differences in metabolism, in C57BL/6J mice.

## Methods

### Clinical study

This study was conducted as an ancillary study to a double‐blind, placebo‐controlled RCT evaluating the efficacy and safety of YKS for preoperative anxiety and prevention of PD (RCT, UMIN000027561^25^, ^30^). Briefly, the study drug (YKS or placebo) was administered for 4 to 8 days before surgery to patients undergoing invasive cancer resection lasting 6 h or longer. The presence of PD was confirmed by trained psychiatrists and researchers, who had been validated for interrater reliability according to DSM‐5 criteria. The study did not meet the prespecified primary endpoint of a 50% reduction in PD.^25^ However, the secondary analysis showed that YKS reduced PD with agitated behavior in patients 75 years or older.^26^ Given the age of patients who exhibited PD in previous studies^25^, ^31^ and the exploratory nature of the present study, we enrolled patients who were 65 years or older who met the criteria of the full analysis set of the RCT (i.e. defined as an eligible population that excluded participants who did not receive protocol treatment and those without a PD assessment) and those who agreed to participate in the secondary study. Additionally, patients were eligible for the current study if they provided plasma samples. The present study explored the metabolites affected by YKS and those involved in the preventive effects of YKS against PD in a primary population of patients 75 years and older who had shown response to YKS.^26^ Peripheral blood was drawn from each patient's artery into a tube containing ethylenediaminetetraacetic acid (EDTA) immediately before surgery under anesthesia^25^ between 9:30 am and 10:30 am. Plasma was stored at 4°C and subsequently isolated at 1500 × g for 10 min within 2 h of blood collection. The isolated plasma was stored at −80°C. All patients in the clinical trial^25^ provided written informed consent for the present study. However, the clinical trial was officially terminated due to the end of the research period, which was approved by the institutional review board (IRB) and ethics committee of the National Cancer Center Japan for this study. The current study was thus additionally approved as an ancillary study by IRB (2019‐267). An option to opt out of the study was provided on the National Cancer Center Japan website. The present study conforms to the provisions of the Declaration of Helsinki.

### Animal study

Male C57BL/6 mice aged 8 weeks and 23 months (Charles River, Kanagawa, Japan) were used as young and aged mice, respectively. The young and aged mice were divided into two groups: a treatment group that received a daily dose of YKS extract at 1 g/kg for 7 days via oral gavage and a control group that received distilled water under the same conditions.

Blood samples were collected from the vena cava under sevoflurane anesthesia using syringes containing EDTA as an anticoagulant. Blood samples were centrifuged under 4°C to separate plasma, which was then stored at −70°C or lower until subsequent analyses. After blood collection, the mice were euthanized, and the brain was extracted and weighed for further analysis. All samples were immediately frozen and stored at −70°C or lower until further analysis.

### Targeted metabolome analysis

Targeted analysis of the metabolites in plasma and brain extract was conducted according to previously established protocols.^28^, ^32^, ^33^ The targeted metabolites, including hydrophilic metabolites, lipid mediators, and phospholipids, were comprehensively measured using the Method Package (Shimadzu, Kyoto, Japan) with liquid chromatography–tandem mass spectrometry (LC–MS/MS) and gas chromatography–tandem mass spectrometry (GC–MS/MS). They were detected and reliably measured in pooled plasma samples. The detailed procedure of LC–MS/MS and GC–MS/MS analysis is shown in the Supplementary Material. For overlapping hydrophilic metabolites in both GC–MS/MS and LC–MS/MS, the one with the lower coefficient of variation (CV) value was adopted based on the results of the QC samples.

Pooled plasma samples were analyzed every five to 10 samples, and the peaks with a stable peak area (CV <30%) in the pooled plasma samples were used for subsequent analysis. An internal standard was added and used for each analysis to correct the MS sensitivity. In addition, the injection volume and sample dilution factor were determined based on prior analysis of the pooled plasma samples to avoid peak saturation. For brain metabolome analysis, ice‐cold methanol was added, and the frozen brain sample was homogenized. After centrifugation, the supernatants were analyzed using the same method as that used for plasma samples.

### Nontargeted metabolome analysis

Nontargeted metabolome analysis explored the YKS‐derived components and metabolites in plasma with LC–MS. The detailed LC–MS analysis and data processing methods are described in the Supplementary Material.

The acquired data underwent waveform processing, peak height calculation, and peak alignment using MS‐DIAL, version 4.9.^34^ The peak heights were normalized using pooled plasma samples, which were analyzed for every 10 samples. First, peaks whose corrected values were >10 times higher in the YKS group compared with the placebo group were identified as YKS‐derived peaks. These selected peak shapes and alignments were then visually checked for accuracy. Metabolite names were assigned according to our in‐house database by matching m/z values and retention times. For peaks of unknown structure, we performed conjugate estimation using mass differences for glucuronide and sulfate conjugates (i.e., DAC‐Met^29^) and structural estimation using MS‐FINDER.^35^

### Data processing and statistical analysis

Processing of metabolomics data was performed using R software, version 4.1 or newer (http://www.r-project.org/). The procedure is summarized in Fig. S1. Peak area and height were normalized according to internal standards in the targeted metabolomics and pooled plasma samples in the nontargeted analysis. In addition, metabolites with normalized area variation in the pooled plasma samples that were > 30% were removed from the detected metabolites because of instability. The plots, charts, and heatmaps were prepared using the R software. Correlation networks were visualized using Cytoscape software, version 3.10 (http://www.cytoscape.org/).^36^

All statistical tests were performed using R software. Our initial exploration with Orthogonal Partial Least Squares Discriminant Analysis (OPLS‐DA) revealed Q^2^ values <0.3, indicating potential overfitting. Therefore, the Wilcoxon signed rank test compared metabolome profiles and visualized them using volcano plots. Spearman correlation analysis evaluated the relationship between plasma and brain metabolites in the mouse study. Differences in characteristics were tested using two‐sided *t* tests or Fisher exact test. A linear model was used as a sensitivity analysis to adjust for the background of intergroup differences above the threshold. The metabolites were assumed to follow a nonnormal distribution in the linear analysis, and a logarithmic transformation was applied. Because our study comprised several exploratory analyses, including an ancillary study of an RCT and animal studies on the effect of YKS, the multiplicity of statistics was not considered. This exploratory study adopted a *P*‐value <0.05 as the basic threshold to discover metabolites involved in PD and YKS. However, in vivo experiments, a *P* value <0.1 was employed to increase sensitivity. Given the exploratory nature of this study, we did not apply a fold‐change threshold for group comparisons.

## Results

### Patient characteristics

**Fig. 1:**
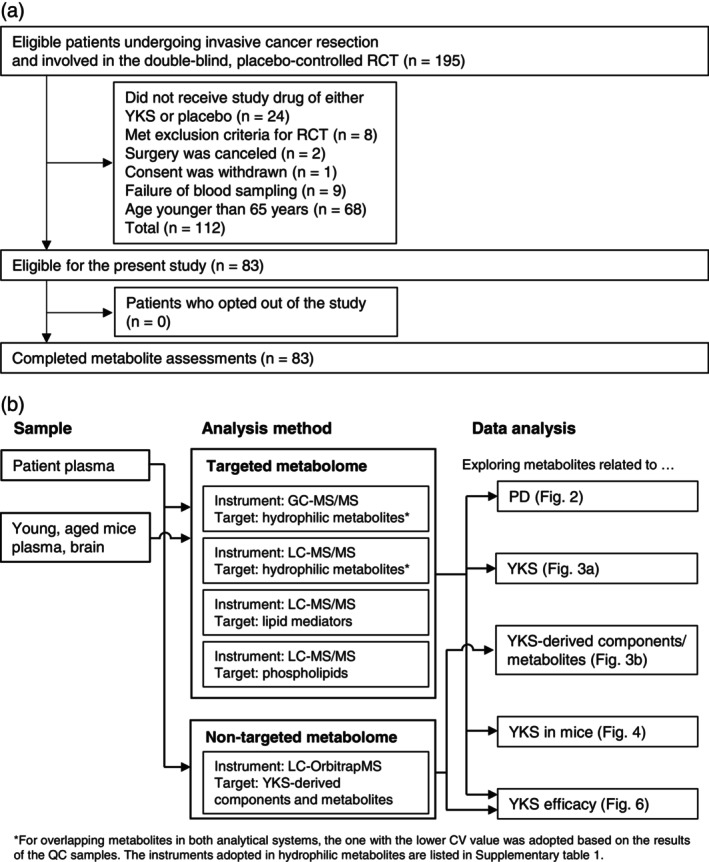
Flowchart of participants and metabolome analysis. (a) Flowchart of the participants. The study was an ancillary study of a double‐blind, placebo‐controlled, randomized controlled trial (RCT) to examine the efficacy and safety of Yokukansan (YKS) for the prevention of postoperative delirium (PD). (b) Flowchart of metabolome analysis. Targeted metabolite analysis using plasma samples collected from patients was performed to identify plasma metabolites associated with PD and the efficacy of YKS (Figs. 2, 3 and 6). Additionally, nontargeted metabolite analysis was performed to identify YKS‐derived metabolites in plasma and to investigate whether YKS‐derived metabolites influence the efficacy of YKS (Figs. 3b and 6). Based on patient results, targeted metabolite analysis was conducted using plasma and brain samples from mice to validate the reproducibility of YKS effects in plasma and to identify metabolites associated with YKS in the central nervous system (Fig. 4). Target analysis measured lipid mediators (liquid chromatography–tandem mass spectrometry [LC–MS/MS]) and phospholipids (LC–MS/MS), and hydrophilic metabolites (gas chromatography–tandem mass spectrometry [GC–MS/MS] and LC–MS/MS). For overlapping hydrophilic metabolites in both GC–MS/MS and LC–MS/MS, the one with the lower coefficient of variation value was adopted based on the results of the QC samples. The instruments adopted in hydrophilic metabolites are listed in Table S1 (patients) and Tables S4–S7 (mice). Nontarget analysis measured YKS‐derived components/metabolites (LC–high‐resolution MS). Further details are described in Supplementary Material. Abbreviation: CV, coefficient of variation.

Eighty‐three patients from the RCT were included in the ancillary study (Fig. 1a). The patient characteristics are presented in Table 1. Notably, in the placebo group, but not the YKS group, the PD group was older than the non‐PD group. Because of our previous findings on the efficacy of YKS,^26^ we examined the effects of YKS in specific age groups. The background information of patients 75 years or older in whom the effects of YKS were examined (*N* = 21) is summarized in Table 2. Among patients 65 years and older, 36 (43.3%) developed delirium, and among those 75 years and older, 12 (57.1%) developed delirium (Tables 1 and 2).

**Table 1:** Patients' characteristics (older than 65 years)

	Placebo	Non‐PD	PD	*P*‐value Non‐PD vs PD	YKS	Non‐PD	PD	*P*‐value Non‐PD vs PD	*P‐*value Placebo vs YKS
Number	**38**	21	17		**45**	26	19		
Male sex, *n* (%)	**25 (65.8)**	11 (52.4)	14 (82.4)	0.09	**28 (62.2)**	13 (50.0)	15 (78.9)	0.07	0.82
Age, mean (SD), y	**71.16 (4.18)**	69.57 (2.69)	73.12 (4.88)	< 0.01	**72.80 (4.37)**	72.65 (3.63)	73.00 (5.31)	0.80	0.09
BMI, mean (SD)	**21.18 (3.13)**	21.19 (2.94)	21.18 (3.44)	0.99	**22.94 (3.26)**	22.97 (3.17)	22.89 (3.47)	0.94	0.02
MMSE score, mean (SD)	**27.42 (2.42)**	27.48 (2.42)	27.35 (2.50)	0.88	**26.76 (2.35)**	26.42 (2.19)	27.21 (2.53)	0.27	0.21
CCI score ≥1, *n* (%)	**13 (34.2)**	5 (23.8)	8 (47.1)	0.18	**17 (37.8)**	8 (30.8)	9 (47.4)	0.35	0.82
Cancer site, *n* (%)				0.10				0.40	0.04
Esophageal	**7 (18.4)**	5 (23.8)	2 (11.8)		**8 (17.8)**	3 (11.5)	5 (26.3)		
Colon	**5 (13.2)**	4 (19.0)	1 (5.9)		**4 (8.9)**	2 (7.7)	2 (10.5)		
Head and neck	**10 (26.3)**	2 (9.5)	8 (47.1)		**4 (8.9)**	1 (3.8)	3 (15.8)		
Hepatobiliary and pancreatic	**14 (36.8)**	9 (42.9)	5 (29.4)		**20 (44.4)**	12 (46.2)	8 (42.1)		
Bone and soft tissue	**0**	0	0		**5 (11.1)**	4 (15.4)	1 (5.3)		
Gynecologic	**2 (5.3)**	1 (4.8)	1 (5.9)		**0**	0	0		
Urinary system	**0**	0	0		**2 (4.4)**	2 (7.7)	0		
Other	**0**	0	0		**2 (4.4)**	2 (7.7)	0		
Education level, *n* (%) More than high school	**24 (63.2)**	13 (61.9)	11 (64.7)	1.00	**22 (48.9)**	12 (46.2)	10 (52.6)	0.77	0.27
ASA class, ≥3, *n* (%)	**11 (28.9)**	5 (23.8)	6 (35.3)	0.49	**9 (20.0)**	7 (26.9)	2 (10.5)	0.26	0.44
CAGE questionnaire, ≥2, *n* (%)	**3 (7.9)**	1 (4.8)	2 (11.8)	0.58	**7 (15.6)**	2 (0.07)	5 (26.3)	0.11	0.33
PS (ECOG), *n*, ≥2	**0**	0	0	1.00	**0**	0	0		1.00

ASA, American Society of Anesthesiologists; BMI, body mass index; CAGE, Cut, Annoyed, Guilty, and Eye; CCI, Charlson Comorbidity Index; ECOG, Eastern Cooperative Oncology Group; MMSE, Mini‐Mental State Examination; PD, postoperative delirium; PS, performance status; SD, standard deviation; YKS, Yokukansan.

**Table 2:** Patients' characteristics (older than 75 years)

	Placebo	Non‐PD	PD	*P*‐value Non‐PD vs PD	YKS	Non‐PD	PD	*P*‐value Non‐PD vs PD	*P*‐value Placebo vs YKS
Number	**6**	1	5		**15**	8	7		
Male sex, n (%)	**5 (83.3)**	0 (0.0)	5 (100.0)	0.17	**7 (46.7)**	3 (37.5)	4 (57.1)	0.62	0.18
Age, mean (SD), y	**78.33 (2.66)**	75.00 (NA)	79.00 (2.35)	0.19	**77.47 (2.47)**	76.50 (1.93)	78.57 (2.70)	0.11	0.49
BMI, mean (SD)	**21.23 (2.43)**	17.70 (NA)	21.94 (1.91)	0.11	**23.75 (4.14)**	23.81 (4.01)	23.69 (4.60)	0.96	0.18
MMSE score, mean (SD)	**26.00 (2.97)**	26.00 (NA)	26.00 (3.32)	1.00	**26.93 (2.52)**	26.38 (2.77)	27.57 (2.23)	0.38	0.47
CCI score ≥1, *n* (%)	**4 (66.7)**	1 (100.0)	3 (60.0)	1.00	**8 (53.3)**	4 (50.0)	4 (57.1)	1	0.66
Cancer site, *n* (%)				1.00				0.33	0.79
Esophageal	**1 (16.7)**	0	1 (20.0)		**2 (13.3)**	0	2 (28.6)		
Colon	**0**	0	0		**2 (13.3)**	1 (12.5)	1 (14.3)		
Head and neck	**2 (33.3)**	0	2 (40.0)		**2 (13.3)**	1 (12.5)	1 (14.3)		
Hepatobiliary and pancreatic	**3 (50.0)**	1 (100.0)	2 (40.0)		**6 (40.0)**	3 (37.5)	3 (42.9)		
Bone and soft tissue	**0**	0	0		**3 (20.0)**	3 (37.5)	0		
Gynecologic	**0**	0	0		**0**	0	0		
Urinary system	**0**	0	0		**0**	0	0		
Other	**0**	0	0		**0**	0	0		
Education level, *n* (%) More than high school	**5 (83.3)**	1 (100.0)	4 (80.0)	1.00	**5 (33.3)**	2 (25.0)	3 (42.9)	0.61	0.06
ASA class, ≥3, *n* (%)	**3 (50.0)**	1 (100.0)	2 (40.0)	1.00	**3 (20.0)**	2 (25.0)	1 (14.3)	1.00	0.29
CAGE questionnaire, ≥2, n (%)	**0**	0	0	1.00	**1 (6.7)**	0	1 (14.3)	0.47	1.00
PS (ECOG), *n*, ≥2	**0**	0	0	1.00	**0**	0	0	1.00	1.00

ASA, American Society of Anesthesiologists; BMI, body mass index; CAGE, Cut, Annoyed, Guilty, and Eye; CCI, Charlson Comorbidity Index; ECOG, Eastern Cooperative Oncology Group; MMSE, Mini‐Mental State Examination; PD, postoperative delirium; PS, performance status; SD, standard deviation; YKS, Yokukansan.

### Plasma metabolome analysis of patients

A total of 199 targeted metabolites (e.g. amino acids, fatty acids, lipid mediators, and phospholipids; Table S1) were detected using the multiple reaction monitoring method (Figure 1b). Analysis of the pooled quality control samples (see Methods) confirmed that these metabolites were stable. In addition to the targeted metabolites, YKS‐derived metabolites in plasma were detected via nontargeted metabolome analysis using high‐resolution mass spectrometry (see below).

### Differences in plasma metabolites between patients with and without PD

**Fig. 2:**
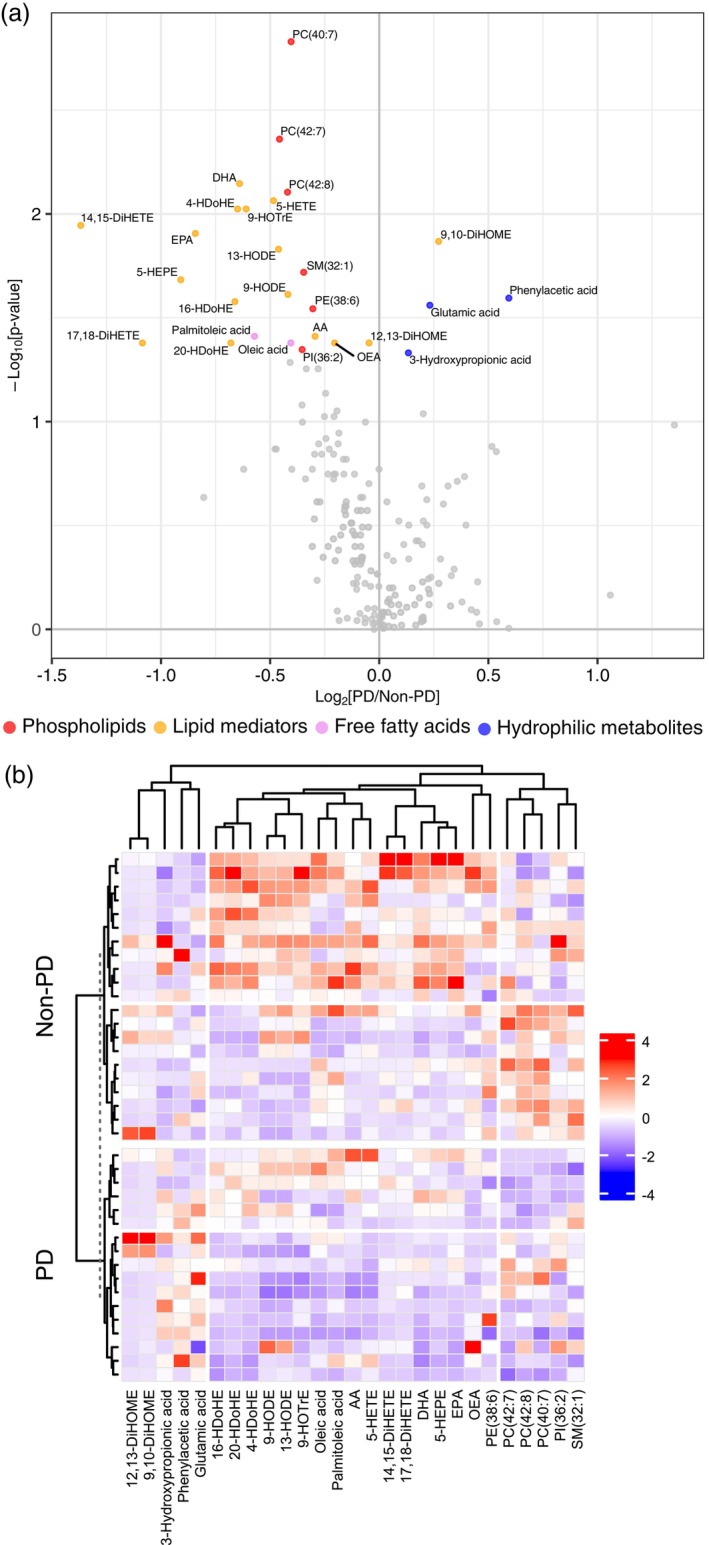
Plasma metabolome profiling of postoperative delirium (PD) and non‐PD patients in the placebo group. Targeted metabolite analysis using plasma samples collected from patients was performed to identify plasma metabolites associated with PD. (a) Volcano plot of the plasma metabolome analysis. Plots were mapped using the log_2_ fold‐change value of PD/non‐PD patients vs the log_10_ *P*‐value obtained from the Wilcoxon signed rank test. The colored points met the criterion of *P* < 0.05 from Wilcoxon signed rank test. Red, yellow, pink, and blue indicate phospholipids, lipid mediators, free fatty acids, and hydrophilic metabolites, respectively. (b) Clustering analysis of plasma metabolites that met the criterion of *P* < 0.05. Data were scaled, and distances were calculated using Spearman correlation coefficient.

To explore the metabolites associated with the development of PD, plasma metabolite profiles measured by the targeted metabolome analysis were compared between patients in the placebo group who did and did not develop PD (*N* = 17 and *N* = 21, respectively). The results of the comparison are shown in Fig. 2a. A total of 27 metabolites were selected. Plasma levels of six phospholipids, 15 lipid mediators, and two free fatty acids were lower and 9, 10‐DiHOME, glutamic acid, phenylacetic acid, and 3‐hydroxypropionic acid levels were higher in the group that developed PD than in the group that did not develop PD. These metabolites were used as variables for clustering based on Spearman correlation coefficient, and the results were visualized as a heatmap (Fig. 2b). The 27 metabolites were classified into three clusters: hydrophilic metabolites, phospholipids, and lipid mediators/free fatty acids. Among those who did not develop PD, a distinct group exhibited higher phospholipid levels, while another group showed higher lipid mediators than others (Fig. 2b). As patients with delirium in the placebo group were older than those in the nondelirium group (Table 1), we conducted a sensitivity analysis to examine validity by adjusting for age. Consistent with the univariate analysis, multiple phospholipids and lipid mediators remained lower in the delirium group (Table S2).

### Effect of YKS administration on the plasma metabolome

**Fig. 3:**
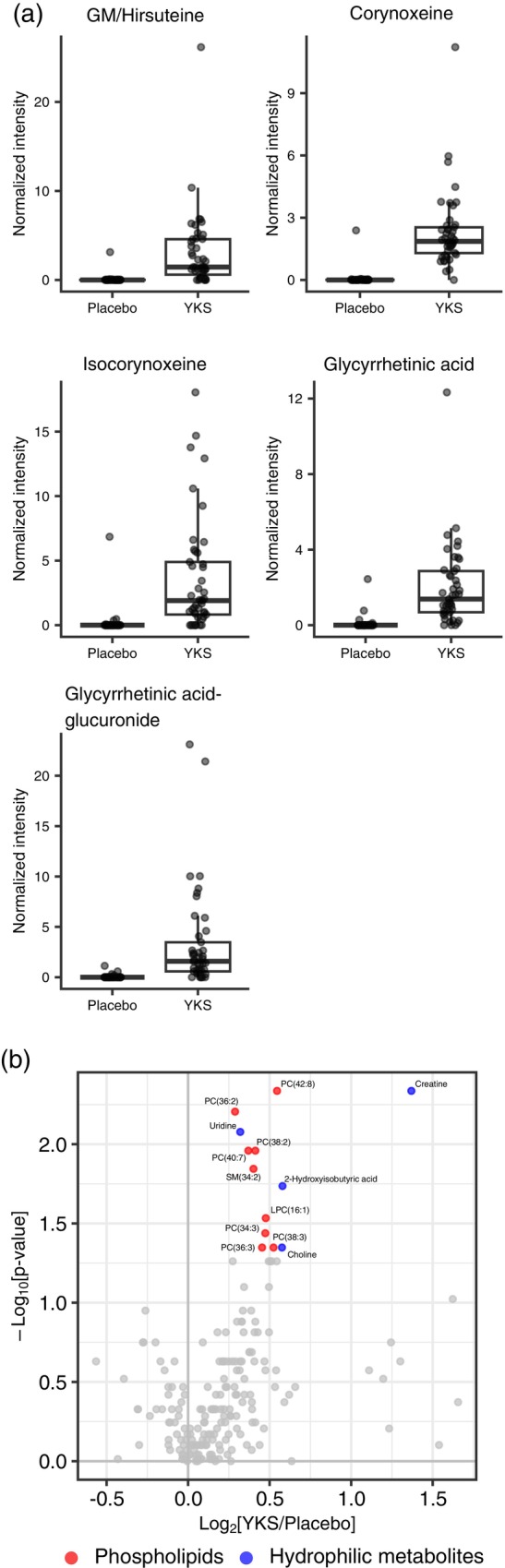
Plasma metabolome profiling in the Yokukansan (YKS) group. (a) Nontargeted metabolite analysis was performed using plasma samples collected from patients and identified the YKS‐derived components and metabolites. Box plots depict the normalized intensities of the YKS‐derived components and metabolites in plasma, with each sample peak height normalized to that of the quality‐control sample. Geissoschizine methyl ether (GM) and hirsuteine are represented as a mixture because the peaks could not be separated. (b) Targeted metabolite analysis was performed using plasma samples collected from patients, and plasma metabolites associated with YKS were identified. Volcano plot comparing the metabolomic profiles between the YKS and placebo groups in patients older than 75 years. The plot corresponds to Fig. 2a.

First, we used a high‐resolution mass spectrometer to explore YKS‐derived components or their metabolites in plasma. We first performed a nontargeted metabolome analysis. Among the peak groups that showed >10 times higher average peak intensities in the YKS group than in the placebo group, a total of 15 YKS‐derived components and metabolites were identified or estimated according to in‐house reference database information (identified: five, estimated: 10; Table S3). Figure 3a shows the five components/metabolites that were considered to be involved in the main pharmacological effects of YKS. All components were rarely detected in the placebo group but were detected in various levels in the YKS group. Among the identified components, geissoschizine methyl ether (GM) and hirsuteine could not be separated by LC. Therefore, the two components are represented as a mixture. In addition, we found 10 metabolites and inferred their structures via MS/MS spectra and DAC‐Met analyses,^29^ which showed that the majority of metabolites were glucuronic and sulfate conjugates.

Next, to evaluate the effect of YKS administration on the metabolites in plasma, we compared plasma metabolome profiles measured by the targeted metabolome analysis between the YKS and placebo groups. When all cases were included, only two metabolites showed a significant difference (Fig. S2), which was not changed when adjusting the difference in body mass index between the YKS group and the placebo group (Table 1). Given previous evidence demonstrating the efficacy of YKS in individuals 75 years or older,^26^ we compared the plasma metabolome between groups in individuals 75 years or older (Fig. 3b). Thirteen metabolites, nine phospholipids, and four hydrophilic metabolites were elevated in the YKS group.

### Mouse plasma and whole brain metabolome analysis

**Fig. 4:**
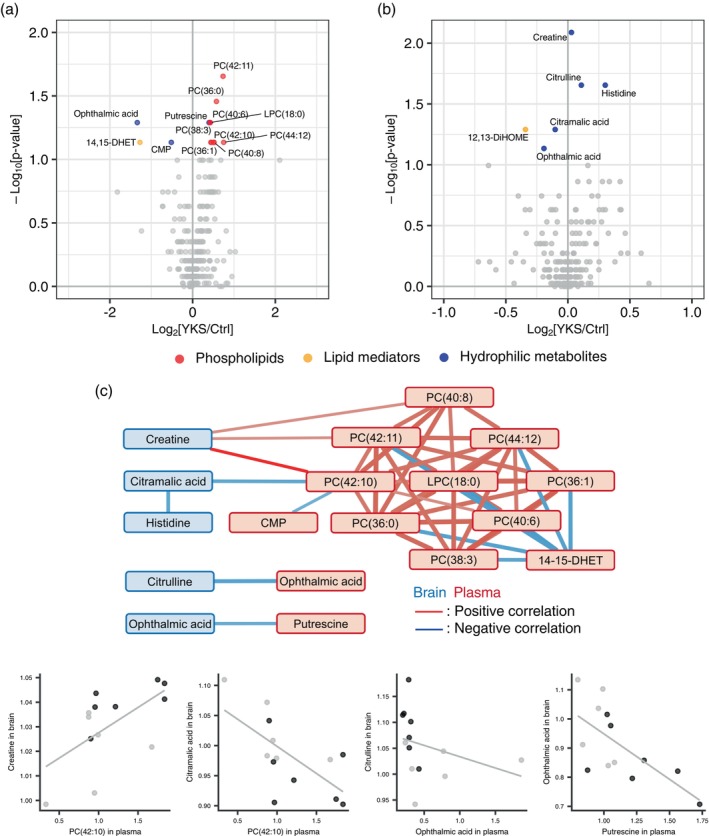
Metabolome analysis of the plasma and brain of aged mice. Targeted metabolite analysis was performed using plasma and brain tissue collected from aged mice and identified metabolites associated with Yokukansan (YKS). (a, b) Volcano plot of plasma (a) and the whole brain (b) depicts the metabolomic differences between the YKS and the control. The plots correspond to that presented in Fig. 2a. The colored plots met the criterion of *P* < 0.1. (c) The correlation network was constructed for the metabolites (*P* < 0.1) and the combination of plasma and brain metabolites in which the absolute correlation coefficient was >0.5 in both the YKS group and all patients. Red and blue nodes represent plasma and brain metabolites, respectively. Red and blue edges represent positive and negative correlations, respectively. The associations between brain and plasma metabolites found in this analysis are represented in the scatter plots. Black and gray circles represent the YKS and control (Ctrl) groups, respectively. Data were collected from *n* = 6 in the YKS group and *n* = 7 in the Ctrl group. Abbreviation: PC, phosphatidylcholine.

We administered YKS or water to 23‐month‐old mice for 7 days and analyzed their blood and brain samples collected the day after final administration. The metabolomic relationship between the changes in plasma and brain metabolites measured by the targeted metabolome analysis was analyzed (Tables S4 and S5). Using a threshold of *P* < 0.1, we selected 13 metabolites in plasma (Fig. 4a) and six metabolites in the brain (Fig. 4b). In plasma, we observed an increase in nine phospholipids in the YKS group. The PC (42:8) and PC (40:7) increases are consistent with clinical findings. In the brain, creatine, citrulline, and histidine increased, whereas citramalic acid, ophthalmic acid, and 12,13‐ DiHOME decreased in the YKS group. When an identical procedure was conducted in young 8‐week‐old mice, plasma and brain metabolite changes were not observed to the same extent as those observed in the aged mice (Fig. S3, Tables S6 and S7). To examine the relationship between the selected metabolites in the plasma and brain, we extracted combinations with absolute Spearman correlation coefficients >0.5 for all cases and exclusively the YKS group and represented them as a correlation network (Fig. 4c). The levels of plasma phospholipids in the YKS group were associated with those of brain creatine, citrulline, and citramalic acid, and the plasma putrescine level also correlated with brain ophthalmic acid level. Scatter plots of these correlations are shown in Fig. 4d.

**Fig. 5:**
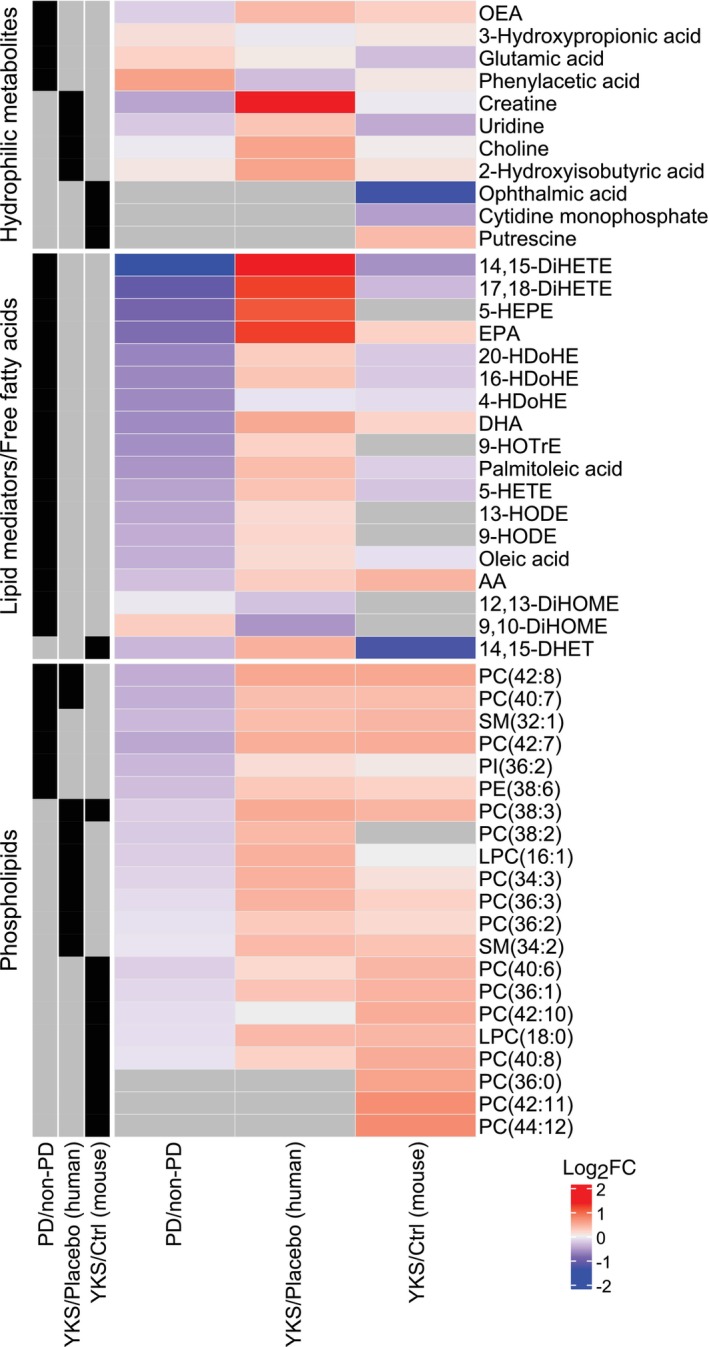
Summary of plasma metabolites in the three comparison groups. The results of the comparisons (postoperative delirium [PD] vs non‐PD in the placebo group, Yokukansan [YKS] vs placebo in patients older than 75 years, and YKS vs control [Ctrl] in aged mice) are summarized as heat maps. The blue and red color gradients indicate the logarithm of the fold‐change in each comparison. Gray indicates undetected metabolites. Candidate metabolites selected to characterize PD and YKS in each comparative analysis are shown in black in the left column.

Figure 5 presents a summary of the results from the above analysis. Most lipids (phospholipids and lipid mediators/free fatty acids) were decreased in the patients with PD. In contrast, the YKS group had higher levels of these lipids, except for 12,13‐DiHOME and 9,10‐DiHOME. In comparison between humans and mice, phospholipid levels were commonly higher in the YKS group, but results for lipid mediators and free fatty acids were inconsistent. No common pattern of change was observed for hydrophilic metabolites.

### Exploration of metabolites involved in response to YKS

**Fig. 6:**
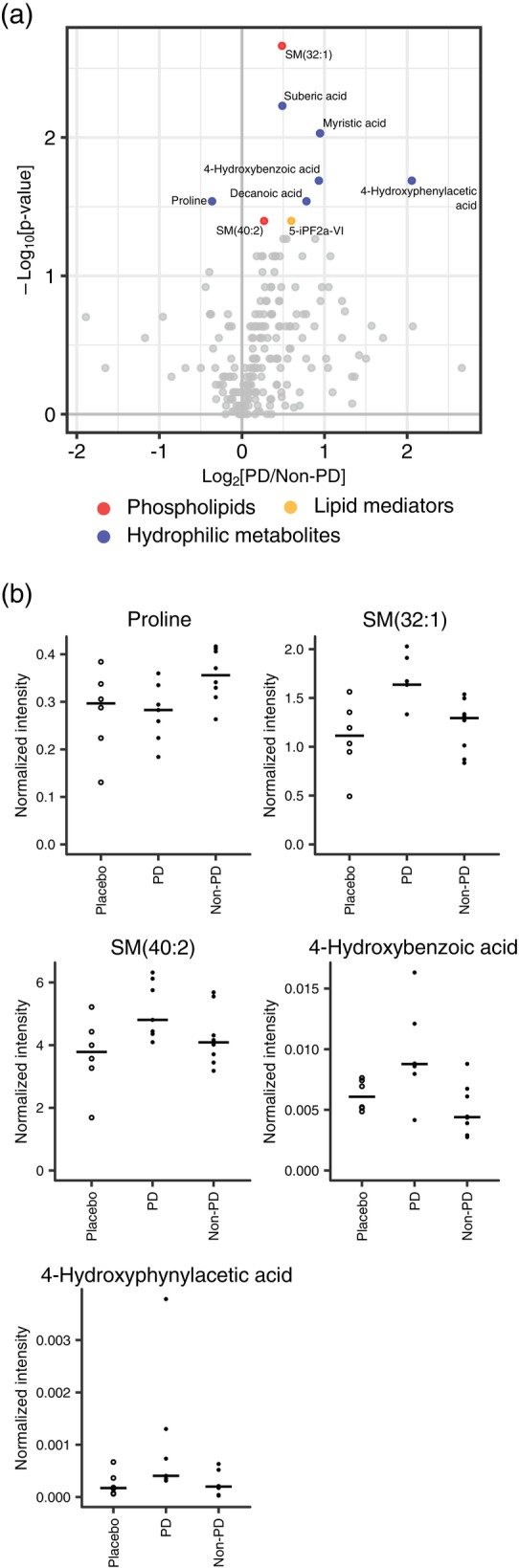
Comparison of plasma metabolites between the occurrence of postoperative delirium (PD) in the Yokukansan (YKS) group. To investigate the response to YKS, we compared targeted and nontargeted metabolite analyses in PD patients and non‐PD patients and examined metabolites associated with the onset of PD in the YKS group. (a) Volcano plot of the plasma metabolites comparing positive and negative PD (patients older than 75 years). The plots correspond to Fig. 2a. (b) Plots of representative metabolites. The crossbar indicates the median. Open and closed circles represent the placebo and YKS groups. Abbreviations: 5‐iPF2a‐VI, (8β)‐5,9α,11α‐trihydroxy‐prosta‐6E,14Z‐dien‐1‐oic acid; SM, sphingomyelin.

We compared the plasma metabolome measured by the targeted and the non‐targeted metabolome analysis between patients with and without PD to identify metabolites associated with the development of PD in the YKS group, which would also offer clues regarding response to YKS. From the volcano plot (Fig. 6a), nine metabolites were selected, which comprised six hydrophilic metabolites, two phospholipids (SM (32:1) and SM (40:2)), and one lipid mediator. Notably, these candidate metabolites did not contain any YKS‐derived components. Proline was the only metabolite that showed an increase in the YKS group among patients who did not develop PD; moreover, its level was higher in this group than in the placebo group (Fig. 6b). We also identified metabolites in the tyrosine/phenylalanine pathway derived from gut bacteria,^37^ 4‐hydroxybenzoic acid, and 4‐hydroxyphenylacetic acid (Fig. 6b).

## Discussion

In the present study, we identified 13 elevated the targeted metabolites in older adult patients aged 75 years or older in whom YKS induced a delirium‐preventive effect. Notably, levels of phospholipids, specifically PC (PC [40:7] and PC [42:8]), in placebo group patients with PD were lower than in those without PD (Fig. 2b, Table S2). Other phospholipids and lipid mediators selected in the analysis showed similar tendencies (Fig. 5). These data suggest a link between phospholipid elevation in the YKS group and PD prevention. In contrast, in patients 65 years or older, the levels of only two metabolites differed significantly between the YKS and placebo groups (Fig. S2). This discrepancy by age aligns with previous findings demonstrating that the efficacy of YKS varies depending on age.^26^ Intriguingly, in the vivo study, the effect of YKS on plasma and brain metabolites was minimal in the young mice (Fig. S3), whereas in aged mice the administration of YKS extract was associated with an increase in plasma phospholipids. In the brain, there was also an increase in creatine, citrulline, and histidine levels, whereas citramalic acid, ophthalmic acid, and 12, 13‐DiHOME levels decreased in the YKS group.

In contrast to the commonly used young mice, aged mice exhibit significant variability due to the accumulation of various environmental factors. By exploiting this variability, our correlation analyses between plasma and brain metabolites revealed associations between plasma phospholipid levels and brain creatine and citramalic acid levels and between plasma ophthalmic acid and putrescine levels and brain citrulline and ophthalmic acid levels (Fig. 4b). The presence of creatine in the brain is associated with cognitive function and neurodegenerative diseases.^38^ Creatine deficiency syndrome is characterized by learning delays, psychiatric disorders, and developmental disabilities, symptoms that partially improve with creatine supplementation.^39^ However, the role of citramalic acid in the brain has not been extensively studied and remains unclear. Citrulline in the brain has been shown in rats to protect against aging‐related oxidative stress.^40^ Although ophthalmic acid, a metabolite related to glutathione, has no known functions in the brain, it is considered a biomarker for liver oxidative stress.^41^ These findings suggest that YKS reduces oxidative stress in the brains of aged mice, and this effect is reflected in plasma phospholipid levels.

We also explored plasma metabolites associated with the development of PD following invasive cancer resection. Our analysis targeting the placebo group revealed that patients who developed PD had lower levels of plasma lipid mediators and phospholipids (Fig. 2a, Table S2), consistent with previous research on cardiac and orthopedic surgeries.^14^, ^16^, ^18^, ^19^ These data suggest that physiological vulnerability to PD is similar among invasive cancer resection and cardiac and orthopedic surgery, validating our metabolomic analysis in this context. A novel insight gained from our work is the distinct lipid profiles of patients who did not develop PD, particularly in the lipid mediator and phospholipid categories (Fig. 2b). These observations suggest that these lipid classes play a specific role in the pathogenesis of PD.

YKS has been extensively studied for its effects in both psychiatric and nonpsychiatric fields in basic studies. YKS is primarily used for treating behavioral and psychological symptoms of dementia (BPSD), but its applications extend to other neurological and pain‐related conditions. Regarding BPSD, YKS has shown efficacy in ameliorating symptoms such as aggressiveness, agitation, and hallucinations in patients with dementia. It acts on multiple neurotransmitter systems, including serotonergic and glutamatergic pathways.^23^, ^42^ In vivo studies on mice have demonstrated that YKS can reduce aggressive behavior induced by social isolation. This effect is linked to the modulation of 5‐HT receptors (HTR2A and HTR3A) expression.^43^ Additionally, YKS improved performance in cognitive tasks in aged and Alzheimer disease model mice.^44^ As described above, YKS has been demonstrated to be effective in the treatment of psychiatric symptoms, and we believe that the same mechanism is at work in the prevention of PD in this study.

Using nontargeted metabolomics, we identified five plasma metabolites derived from YKS (Fig. 3a). Previous pharmacokinetic studies in healthy adults have documented the blood concentration profiles of GM, hirsuteine, and glycyrrhetinic acid (GA) post‐YKS administration.^45^ GM and hirsuteine, alkaloid components from the Uncaria hook, are known for their 5‐HT receptor activity, contributing to the pharmacological effects of YKS.^46^ The pharmacokinetic half‐lives (*t*_1/2_) of GM and hirsuteine are 1.72 h and 2.47 h, respectively, and the time to reach maximum concentration (*t*_max_) are 0.5 h and 1.0 h, respectively. Given our sampling time of approximately 12 h after the final dose, we anticipated that these Uncaria hook alkaloids would be undetectable. However, GM/hirsuteine, corynoxeine, and isocorynoxeine were detected in the YKS group with significant variability in peak intensities, which was possibly due to decreased hepatic clearance in older adults. GA, a metabolite of glycyrrhizin derived from licorice, is absorbed following deconjugation by gut bacteria. It promotes neuroplasticity and anti‐inflammatory effects, likely affecting various neurotransmitters.^22^, ^23^ We observed that GA had a longer *t*_1/2_ and *t*_max_ of 11 h and 8 h, respectively, and was thus detectable in our study. Although these components are involved in the pharmacological action of YKS, no differences were found between those in the YKS group who did and did not develop PD (Fig. 6a). This observation suggests that the effectiveness of YKS cannot be predicted purely by the concentration of components in the blood; rather, complex factors are likely at play.

Japanese Kampo medicine is prescribed for patients who fulfill the Kampo diagnosis known as “*sho*,” with the condition and symptoms varying among patients. Because the concept of Kampo diagnosis has not yet been fully elucidated, we compared plasma metabolite profiles of the YKS group between patients who did and did not develop PD (Fig. 6) to gain further insight into the Kampo diagnosis. Both 4‐hydroxybenzoic acid and 4‐hydroxyphenylacetic acid showed a group difference. These metabolites possess antioxidant properties owing to the metabolism of tyrosine and polyphenols by gut bacteria.^37^, ^47^, ^48^, ^49^ Furthermore, they increase following the administration of selective 5‐HT reuptake inhibitors in patients with major depressive disorder.^50^ These data suggest a link between the gut environment, PD, and response to YKS. We also identified two types of SMs (SM [32:1] and SM [40:2]). Several reports have confirmed a relationship between blood SM concentrations and brain function.^51^, ^52^ One study demonstrated that SM concentrations increase with age and may be a risk factor for Alzheimer disease. In contrast, another report observed that patients with Alzheimer disease who have high SM concentrations exhibit slower deterioration of cognitive function. Therefore, it is necessary to conduct further clinical and basic research to clarify the relationship between blood SM concentrations and brain function.

The limitations of this study include the small sample size of patients older than 75 years and the use of an exploratory analysis without multiple comparison corrections or adjustment of patient background data. Although a multivariate analysis such as OPLS‐DA was considered to select metabolites contributing to the discrimination of the two groups, there were concerns about overlearning in any of the analyses (Q2 values from cross‐validation in OPLS‐DA were <0.3). The YKS and placebo groups were randomly assigned in a double‐blind RCT. Concerning the background of the delirium group and the nondelirium group, or the YKS group and the placebo group, it was considered that there were few differences between the two groups that would have a major impact on the interpretation of the results of the metabolome analysis (Tables 1 and 2). Therefore, univariate analyses were primarily performed in this study. In comparing the PD and non‐PD patients 65 years or older, the PD group was older than the non‐PD group, which may have affected the characteristics of the metabolites detected in the PD group. In addition, there was a higher body mass index in the YKS group than in the placebo group, which may have led to a lower estimate of YKS‐derived components. To confirm the validity of the results, we adjusted these covariates as a sensitivity analysis for the metabolites we identified. However, this did not affect our findings that YKS may contribute to the prevention of PD via an increase in phospholipids. Because the number of patients 75 years or older was low, we could not examine the impact of background factors. However, we did not find significant differences in background factors between PD and non‐PD patients (Table 2). As a strength of this study, we ensured the internal validity and generalizability of the study by conducting an ancillary study to a double‐blind, placebo‐controlled RCT. Specifically, in addition to strict eligibility and exclusion criteria, the diagnosis of delirium (which ensured interrater reliability for PD diagnosis among trained psychiatrists or research assistants who learned clinical psychology) and the regulation of concomitant use of other herbal medicines and benzodiazepines enabled the high quality of the study. For the metabolome analysis, blood samples were collected at a fixed time immediately before surgery from patients in a fasted state to eliminate the influence of diet and diurnal variation. Nevertheless, our findings require validation via clinical studies with larger samples and basic research to elucidate the specific mechanisms underlying YKS effects. In particular, clinical research will be required to verify whether administering YKS to elderly patients with cancer with low plasma phospholipid levels increases phospholipid levels and prevents PD. Additionally, correlating preadministration blood sample profiles with diagnostic outcomes is necessary to understand Kampo diagnosis comprehensively.

Our findings offer further insight into the mechanism underlying the efficacy of YKS in preventing PD in older adults and the molecules related to drug responsiveness. Additional analyses will enable a better understanding of Japanese Kampo medicines' complex mechanisms and utility.

## Disclosure statement

This study was supported by Research on Development of New Drugs, the Japan Agency for Medical Research and Development (AMED, No. JP21ak0101160), and Tsumura & Co. The metabolome analyses and the animal study were performed by Tsumura & Co. The funders had no role in any aspect of study design, clinical data collection and management, data analysis and interpretation, or manuscript submission. Although the original ProD trial was supported by Tsumura & Co., investigators' professional judgments in the study were not influenced by Tsumura & Co. KO and TN were employed by Tsumura & Co. TY was granted consulting fees from Tsumura & Co. HM, on behalf of the Department of Psycho‐oncology, National Cancer Center Japan, and YU also received funding from Tsumura & Co.

## Author contributions

Conceptualization, R.S., K.O., and Y.U.; Methodology, K.O., T.N., and M.S.; Software, R.S., K.O.; Formal Analysis, R.S., K.O. and T.Y.; Investigation, K.O. and T.N.; Resources, R.S., S.M., T.S., M.E., H.D., S.Y., Y.K., A.K., M.I., Y.M., K.A., T.U., H.M. and Y.U.; Data Curation, R.S., S.W., and K.S.; Writing – Original Draft Preparation, R.S. and K.O.; Writing – Review & Editing, All authors; Visualization, R.S. and K.O.; Supervision, M.S., K.A., H.M. and Y.U.; Project Administration, Y.U.; Funding Acquisition, Y.U.

## Supporting information


**Figure S1.** Flowchart of metabolome data processing.
**Figure S2.** Plasma metabolome profiling of the Yokukansan (YKS) group (patients).
**Figure S3.** Metabolome analysis of the plasma (left) and brain (right) of young mice.

Data S1.

Data S2.

## Data Availability

Data may be provided following consultation with the corresponding author.
