# Supplementary material for: Metabolome Profiling of Yokukansan in Preventing Postoperative Delirium in Elderly Cancer Patients: A Reverse Translational Study
Source: Psychiatry Clin Neurosci. 2025 Aug 5;79(10):685–96. doi: 10.1111/pcn.13875 (PMC12498125; doi:10.1111/pcn.13875)
Supplement: Supplementary file 1 — Figure S1. Flowchart of metabolome data processing. Figure S2. Plasma metabolome profiling of the Yokukansan (YKS) group (patients). Figure S3. Metabolome analysis of the plasma (left) and brain (right) of young mice. [file PCN-79-685-s001.pdf]

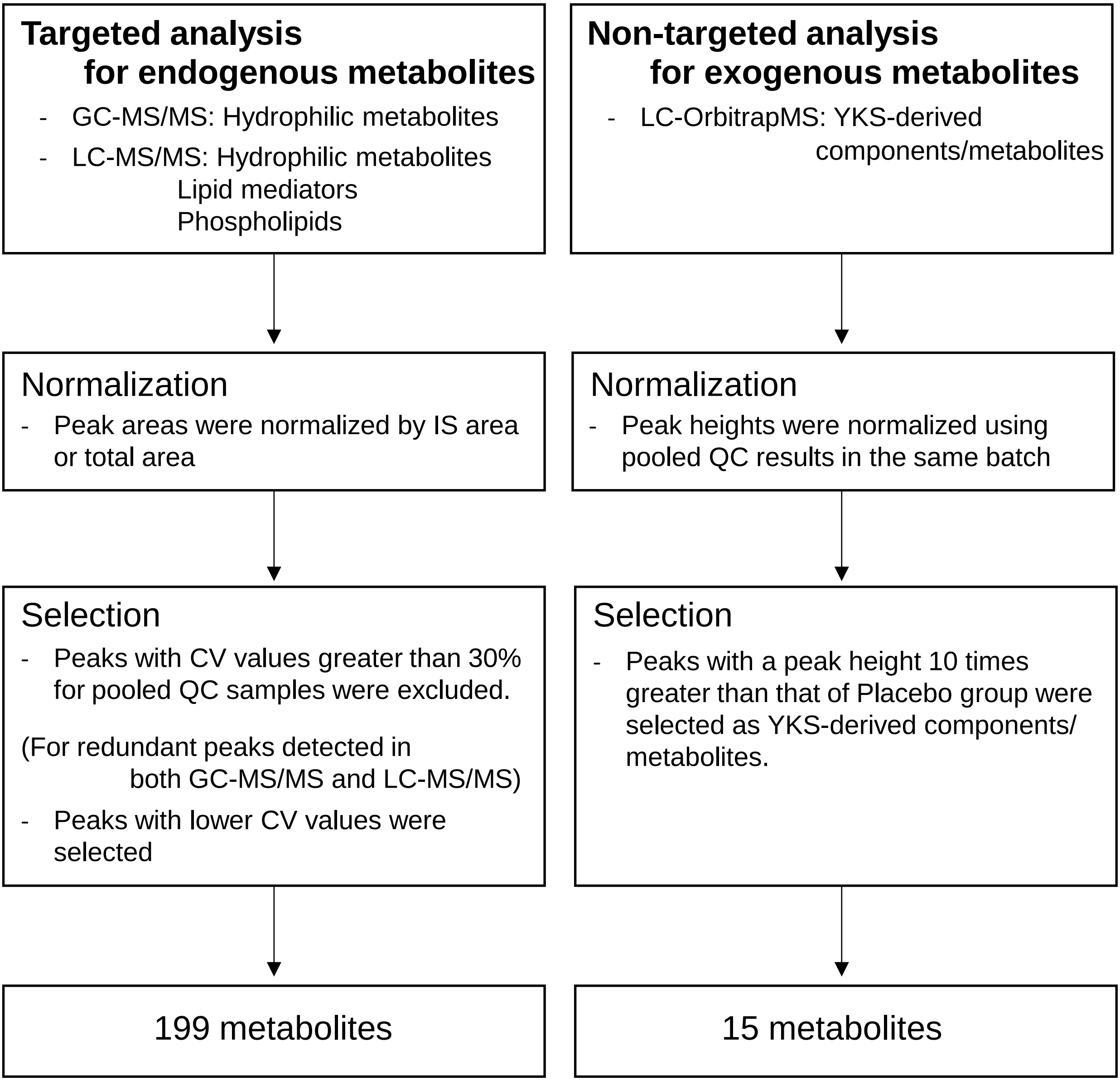

Supplementary Figure 1. Flowchart of metabolome data processing
Plasma metabolome analysis was performed in targeted and non-targeted formats. After GC-MS/MS and LC-MS/MS analyses, normalization and peak selection were conducted. As a result, total 199 endogenous metabolites and 15 YKS-derived components/metabolites were used for further analyses.

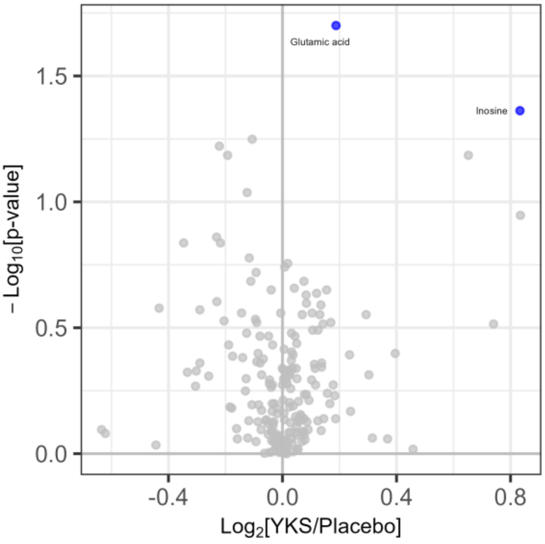

Supplementary Figure 2. Plasma metabolome profiling of the Yokukansan (YKS) group (patients aged over 65 years). Volcano plot comparing the metabolomic profiles between the YKS and placebo groups in patients aged over 65 years. This plot corresponds to Figure 2a.

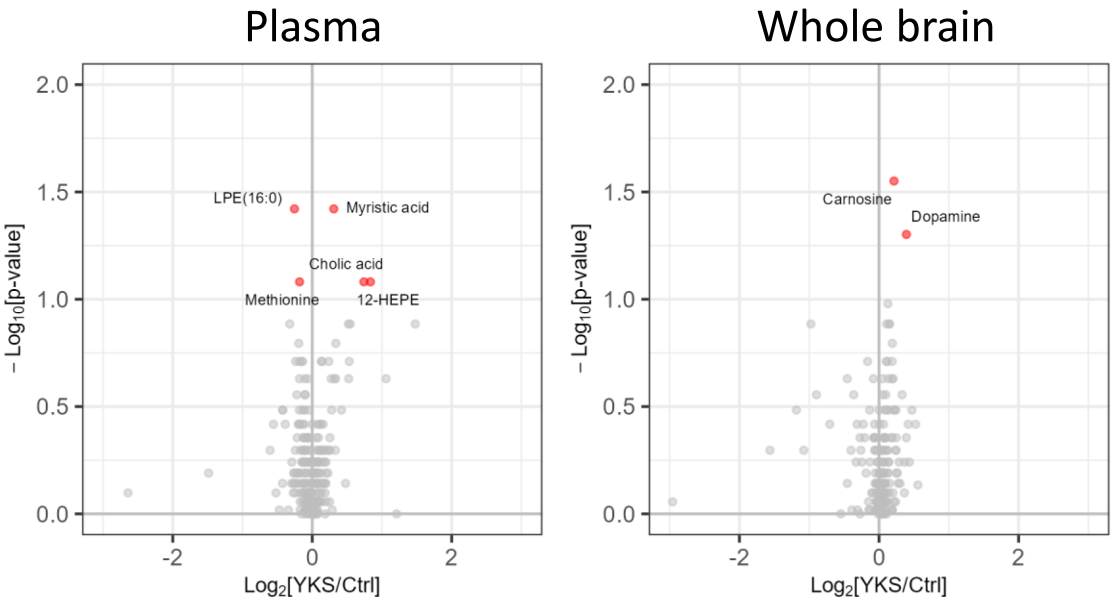

Supplementary Figure 3. Metabolome analysis of the plasma (left) and brain (right) of young mice. Volcano plot of plasma (a) and the whole brain (b). Plots were mapped using the log2 fold-change value of Yokukansan (YKS)/control (Ctrl) versus the log10 *p*-value obtained from the Wilcoxon signed-rank test. The colored points met the criterion of $p < 0.1$. Data were collected from n = 8 in both the YKS and Ctrl groups. HEPE, hydroxy-eicosapentaenoic acids; LPE, lyso-phosphatidylethanolamine
