# Supplementary material for: Metabolome Profiling of Yokukansan in Preventing Postoperative Delirium in Elderly Cancer Patients: A Reverse Translational Study
Source: Psychiatry Clin Neurosci. 2025 Aug 5;79(10):685–96. doi: 10.1111/pcn.13875 (PMC12498125; doi:10.1111/pcn.13875)
Supplement: Supplementary file 3 — Data S2. [file PCN-79-685-s003.docx]

**Supplementary Material**

| Analysis name (data) | | Metabolite class | | Instrument | |
| --- | --- | --- | --- | --- | --- |
| Targeted metabolome analysis (Supplementary Table 1) | | Hydrophilic metabolites* | | GC-MS/MS | |
|  |  | Hydrophilic metabolites* | | LC-MS/MS | |
|  |  | Lipid mediators | | LC-MS/MS | |
|  |  | Phospholipids | | LC-MS/MS | |
| Non-targeted metabolome analysis (Supplementary Table 2) | | YKS-derived components/metabolites | | LC-Orbitrap | |
| *For overlapping metabolites in both analytical systems, the one with the lower CV value was adopted based on the results of the QC samples. | | | | | |
|  | | | | | |
| Figure | Sample and matrix | | Analysis method | |  |
| Figure 2 | Patient plasma  (Placebo, > 65 y, PD (n = 17) vs. Non-PD (n = 21)) | | Targeted | |  |
| Figure 3a | Patient plasma  (> 65y, YKS (n = 45) vs. Placebo (n = 38)) | | Non-targeted | |  |
| Figure 3b | Patient plasma  (> 75y, YKS (n = 15) vs. Placebo (n = 6)) | | Targeted | |  |
| Figure 4 | Mouse plasma and brain  (Aged, YKS (n = 6) vs. Control (n = 7), Young, YKS (n = 8) vs. Control (n = 8)) | | Targeted | |  |
| Figure 6 | Patient plasma  (YKS, > 75y, PD (n = 7) vs. Non-PD (n = 8)) | | Targeted and Non-targeted | |  |

***Targeted metabolome analysis***

***GC-MS/MS metabolome analysis for hydrophilic metabolites***

In the GC-MS/MS analysis, 50 μL of plasma or brain extract was mixed with 150 μL of water and 800 μL of acetonitrile containing 10 μL of 0.5 mg/mL 2-isopropylmalic acid (Sigma–Aldrich, St. Louis, MO, USA) for 30 min at 37°C. The supernatant was transferred to a vial. The analysis was performed using a method based on the principle of the reference [1], [2] and automated derivatization system SGI-M100 system (AiSTI SCIENCE, Wakayama, Japan). In this system, extracted samples were loaded on an ion-exchange cartridge SPE. The target metabolites were retained in the SPE; derivatization was performed by methoxyamine/pyridine and *N*-methyl-*N*-(trimethylsilyl) trifluoroacetamide, which were directly added sequentially on the SPE. The derivatized sample was subjected to measurement by GC-MS/MS.

GC-MS/MS analysis was performed using a GCMS-TQ8040 (Shimadzu, Kyoto, Japan) system with a fused silica capillary column (BPX5: 30 m × 0.25 μm; film thickness, 0.25 μm; SGE, Melbourne, Australia). The analytical condition, chromatogram acquisition, and waveform processing are summarized in the reference [1]. Peak selection and integration were carried out using the Traverse MS software (Reifycs Inc., Tokyo, Japan). The peak intensity of each quantified ion was calculated and normalized to that of 2-isopropylmalic acid, which was used as an internal standard.

***LC-MS/MS metabolome analysis for hydrophilic metabolites***

Hydrophilic metabolites in the plasma or brain extract were also analyzed using LC-MS/MS. Specifically, 10 μL of sample was mixed with 500 μL of methanol containing 20 μM 2-morpholinoethanesulfonic acid and 250 μL of water. The supernatant (600 μL) was transferred to a new tube containing 400 μL of chloroform. Approximately 10 min after mixing, the upper layer (water layer, 200 μL) was transferred to an Amicon Ultra-0.5 mL centrifugal filter (Merck KGaA, Darmstadt, Germany) and filtration was carried out. Following lyophilization of the filtrate, the extracted metabolites were reconstituted with 50 μL of water. Subsequently, LC-MS analysis was performed.

The LC-MS/MS system consisted of two LC-30AD pumps, a SIL-30AC auto-sampler, a CTO-20A column oven, a CBM-20A system controller, and a triple quadrupole mass spectrometer LCMS-8050 (Shimadzu). A pentafluorophenylpropyl column (Discovery HSF5-3, 2.1 mm ID, 150 mm L, 3 μm, Merck KGaA, Darmstadt, Germany) was used for chromatographic separation. Chromatogram acquisition, detection of mass spectral peaks, and their waveform processing were performed using the Labsolutions LCMS software, and LC/MS/MS Method Package for Primary Metabolites (Shimadzu). Peak selection and integration were carried out using the Traverse MS software. The peak intensity of each quantified ion was calculated and normalized to that of 2-morpholinoethanesulfonic acid, which was used as an internal standard.

Some hydrophilic metabolites were targeted by both LC and GC-MS/MS. In this case, we selected the more stably measured metabolites by comparing the CV values of the pooled QC samples.

***LC-MS/MS metabolome analysis for lipid mediators***

Lipid mediators in plasma and brain extract were measured using LC-MS/MS according to the method described by Kitagawa et al. [1]. Briefly, 200 μL of sample was mixed with 1 mL of methanol and the internal standard mixture. The supernatant was diluted with 4 mL of 0.1% formic acid in water. The mixture was loaded onto a preconditioned solid-phase extraction cartridge (10 mg/1 mL; STRATA-X, Phenomenex, Torrance, CA, USA). The cartridge was washed with 0.1% formic acid and 15% ethanol (1 mL of each). Lipids were eluted with 250 μL of 0.1% formic acid in methanol, and the eluent was evaporated using a vacuum evaporator (CC-105 and TU-1000; TOMY SEIKO CO., Tokyo, Japan) and reconstituted in 20 μL of methanol. Then, LC-MS/MS analysis was performed.

Chromatogram acquisition, detection of mass spectral peaks, and their waveform processing were performed using the Labsolutions LCMS software, and LC/MS/MS Method Package for Lipid Mediators (Shimadzu). Kinetex C8 (150 × 2.1 mm I.D., 2.6 μm particle size, Phenomenex) was used for chromatographic separation. The peak areas of each quantified ion were calculated and normalized to those of an internal standard mixture containing tetranor-PGEM-d6, TXB2-d4, PGE2-d4, PGD2-d4, LTC4-d5, LTB4-d4, 5-HETE-d8 and 15-HETE-d8 (0.5 ng/μL each), oleoylethanolamide-d4 (0.25 ng/μL), and arachidonic acid-d8 (10 ng/μL) in methanol. Peak selection and integration were carried out using the Traverse MS software (Reifycs Inc.).

***LC-MS/MS metabolome analysis for phospholipids***

Phospholipids in plasma and brain were measured using LC-MS/MS. 10 μL of sample was mixed with 1 mL of 0.1% formic acid/methanol. The supernatant was transferred into the vial. Then, LC-MS/MS analysis was performed.

Chromatogram acquisition, detection of mass spectral peaks, and their waveform processing were performed using the Labsolutions LCMS software, and LC/MS/MS MRM Library for Phospholipid Profiling (Shimadzu). Phenomenex Kinetex C8 (150 mm × 2.1 mmI.D., 2.6 μm) was used for chromatographic separation. The MRM transitions to determine phospholipid classification were used in this study. The peak areas of each quantified ion were calculated and normalized to the total area. Peak selection and integration were carried out using the Traverse MS software.

***Non-targeted metabolome analysis***

Non-targeted metabolome analysis was performed using 40 µL of plasma, to which 120 µL of methanol was added for protein precipitation. The samples were then mixed thoroughly and centrifuged at 22,000 × g for 3 minutes. The supernatant was subsequently concentrated using a vacuum evaporator and reconstituted into 40 µL of 0.1% formic acid aqueous solution. After centrifugation at 22,000 × g for 3 minutes, the clear supernatant was collected for LC-MS analysis.

Chromatographic separation was carried out on a Vanquish LC system (Thermo Fisher Scientific, Waltham, MA) using an Acquity HSS-T3 column (2.1 mm internal diameter, 150 mm length, and 1.8 µm particle size; Waters, Milford, MA). The mobile phase consisted of 0.1% formic acid in water (A) and acetonitrile (B) using a gradient elution at a flow rate of 0.2 mL/min. The gradient profile was as follows: 0 minutes, 100% A; 30 minutes, 2% A; 35 minutes, 2% A; 35.1 minutes, 100% A; and maintained at 100% A until 45 minutes. The injection volume was 20 µL.

Mass spectrometric detection was performed using an Orbitrap ID-X mass spectrometer equipped with a heated electrospray ionization ion source. Data-dependent acquisition was used to acquire MS/MS spectra, and each sample was analyzed using both positive and negative ion modes.

**References**

[1] H. Kitagawa *et al.*, “Data on metabolic profiling of healthy human subjects’ plasma before and after administration of the Japanese Kampo medicine maoto,” *Data Brief*, vol. 22, pp. 359–364, Feb. 2019.

[2] E. Takeo, R. Sasano, S. Shimma, T. Bamba, and E. Fukusaki, “Solid-phase analytical derivatization for gas-chromatography-mass-spectrometry-based metabolomics,” *J. Biosci. Bioeng.*, vol. 124, no. 6, pp. 700–706, Dec. 2017.
